# The Presence of Conspecific Decoys Enhances the Attractiveness of an NaCl Resource to the Yellow-Spined Locust, *Ceracris kiangsu*

**DOI:** 10.1673/031.011.0145

**Published:** 2011-04-10

**Authors:** Hai-Ping Yu, Zhi-Tian Wang, Kai Xiao, Lin Shao, Guo-Qing Li

**Affiliations:** Department of Entomology, Key Laboratory of Monitoring and Management of Plant Diseases and Pests, Ministry of Agriculture, Nanjing Agricultural University; Nanjing, China, 210095; ^†^ These authors contributed equally to the research.

**Keywords:** decoy, NaCl bait, feeding, aggregations

## Abstract

Adults of the yellow-spined bamboo locust, *Ceracris kiangsu* Tsai (Orthoptera: Oedipodidae), aggregate and gnaw at human urine-contaminated materials, a phenomenon termed puddling. Several urine-borne chemicals, including NaCl, are known to stimulate adult *C. kiangsu* to consume filter paper. Because in nature *C. kiangsu* adults may use cues to locate puddling resources, we tested the influence of conspecific decoys (dried *C. kiangsu*) on foraging and consumption of 3% NaCl—treated filter paper. In a two—choice test experiment in the laboratory, female adults showed no preference for filter papers (not treated with NaCL) with or without decoys. In contrast, *C. kiangsu* females consumed significantly more NaCl—treated filter paper on which conspecific decoys were attached than those without decoys in both the laboratory and in a bamboo forest. When the bait was changed to 3% NaCl plus the insecticide bisultap, significantly more *C. kiangsu* were killed in the bamboo forest when decoys were present, however the results were not significant when the experiment was done in the laboratory. Hence, moving towards conspecifics seems to facilitate NaCl resource foraging in *C. kiangsu*, suggesting that the presence of conspecifics promotes feeding on puddling resources.

## Introduction

Sodium is important in insect development and reproduction. For example, it aids the uptake of amino acids (Wolfersberger 2000), maintains osmotic pressure and stabilizes cell volume, and stimulates the neuromuscular system (Arms et al. 1974). Sodium concentration is very low in most terrestrial plants (Blair-West et al. 1968, Smedley and Eisner 1995), and herbivores may expend considerable energy to find and harvest sodium. Some butterflies, moths, flies, honeybees, ants, and cicadellids are known to search for potential salt sources, such as moist ground, perspiration, tears, excrements, or rotten fish (Shen et al. 2009; Molleman 2010). This phenomenon has been termed puddling, and some insect species, such as the notodontid moth (Smedley and Eisner 1995), some ants (Kaspari et al. 2008), and Mormon crickets (Simpson et al. 2006) are known to puddle to collect sodium.

Adults of the yellow-spined bamboo locust, *Ceracris kiangsu* Tsai (Orthoptera: Oedipodidae), are known to visit excrements and perspiration, and feed on urine— and sweat—soaked materials, especially on hot summer days (Hilgartner et al. 2007, Shen et al. 2009, Molleman 2010). Human urine, especially when incubated, can stimulate the adults to consume filter paper in the laboratory. Consumption of the filter paper can also be stimulated by several urine-borne chemicals, including NaCl, NaH_2_PO4, Na_2_SO_4_, KCl, NH_4_Cl and NH_4_HCO_3_. Among them NaCl has been shown to be a strong phagostimulant (Shen et al. 2009).

Because animal excrement and perspiration end products are usually rare and patchily distributed in nature, *C. kiangsu* adults may use cues, such as visual and chemical stimuli to locate puddling resources. The main chemicals in fresh human urine include inorganic salts and CO(NH_2_)_2_ (Putnam 1971). When human urine is incubated and CO(NH_2_)_2_ decomposes, NH_4_HCO_3_ becomes a rich nitrogenous compound (Kirchmann and Pettersson 1994). Previous studies (Shen et al. 2009) have shown that CO(NH_2_)_2_ acted as a repellent and NH_4_HCO_3_ as an attractant/arrestant, demonstrating that olfactory cues emitted by human urine were important for *C. kiangsu* adults searching for puddling resources.

Adult *C. kiangsu* exhibit joining behavior and form gregarious bands during their lifetime (Li et al. 1998; Zhang et al. 2007). The joining behavior of adult *C. kiangsu* may serve to increase individual fitness in several ways. The first benefit may be to lower per capita predation risk by a dilution effect as other insect species (Sillén-Tullberg 1988; Gamberale and Tullberg 1998; Prokopy et al. 2000; Burger and Gochfeld 2001; Prokopy and Roitberg 2001). Second, joining an aggregation can synchronize breeding (Clayton 1978) and/or enhance attractiveness to potential mates (Chapman et al. 1986; Cade and Cade 1992; Muller 1998). Individuals can also find rare and patchily distributed resources by moving towards conspecific—occupied resources (Arms et al. 1974; Beck et al. 1999; Otis et al. 2006).

Therefore, it is of interest to test whether the presence of conspecifics has any effect on NaCl resource foraging in *C. kiangsu* adults. We also performed some tests in the laboratory and in a bamboo forest with dried locusts as decoys to test the question of whether the presence of conspecifics promotes feeding on puddling resources.

## Materials and Methods

### Insects, chemical and insecticide

*Ceracris kiangsu* eggs were collected from soil near a giant bamboo, *Phyllostachys edulis* (Carriere) Houz (Poales: Poaceae) forest at Nanjing (32.0N, 118.5E), Jiangsu Province, China, March 2008. The nymphs were reared in fabric cages (100×100×150 cm) and fed with stunted black bamboos, *Phyllostachys nigra* (Lindley) Munro, planted in pots. The bamboo plants were changed every day. Males and females from the same batch (< 7 days old) were used in experiments to eliminate variability in physiological status.

To separate the roles of visual and chemical cues, decoys were prepared from males and females (< 7 days old). These adults were killed by freezing, mounted on insect pins with the wings folded, and dried. Care was taken that the pinned decoys closely resembled the natural posture of puddling individuals.

Sodium chloride (NaCl) was purchased from Shantou Xilong Chemical Factory and had a purity of 99%. An 18% aqueous solution of the insecticide, bisultap [thiosulfuric acid, s,s′-(2-(dimethylamino) -1,3- propanediyl) ester, disodium salt], was obtained from Hefei World Chemical Industry Co. LTD, http://hwcic16128.en.china.cn/.

### Two-choice attraction test in the laboratory

Small fabric covered cages with aluminum frames (50×50×50 cm) were used for two-choice tests similar to the method described previously by Shen et al. (2009). In fabric cages, two clean pieces of filter paper (Whatman) (24×50 cm) were used to cover two halves of the floor, leaving a gap of 2 cm between the two. On one piece of the filter paper, a male and a female conspecific decoy were fastened with odorless paste, and the other filter paper was left empty. Our previous results showed that males and females had similar sodium feeding behavior (Shen et al. 2009). To simplify the experiment, here we used females only. Four hours after dawn, one live female was introduced into a cage, and her settling position (either filter paper with or without conspecifics, or fabric) was recorded after 30 minutes. The experiment was replicated 50 times with 50 clean cages. In order to avoid any position effect, the relative position of treatment and control filter papers was switched for each replicate.

### Feeding tests

Tests were carried out both in the laboratory and in a bamboo forest. Filter papers (15 × 5 cm in the laboratory tests, 20 × 20 cm in bamboo forest tests) were immersed for 5 seconds in a 3% NaCl aqueous solution, a concentration similar to that in human urine, and allowed to dry for two hours and then conspecific decoys, with a male/female ratio of 1:1, were attached to half of the NaCl—treated filter papers using odorless paste. Two were attached for the laboratory tests, and four in bamboo forest tests.

Cages (50×50×50 cm) described above were used in the laboratory test. Four hours after dawn, four NaCl—treated filter papers, differing in the presence or absence of conspecific decoys, were fixed on four aluminum frames within the fabric screen cage with steel wire, each filter paper was near but not attached to the ceiling. In each test cage, only one female was introduced to avoid any female—female interactions. Six hours later, the filter papers were collected, and the consumed areas were measured by placing the filter papers on squared paper and estimating the missing area in square millimeters. The test was replicated 45 times with 45 clean cages. In order to avoid any position effect, the relative position of the filter papers with or without decoys was switched for each replicate.

The field tests were conducted as described previously by Shen et al. (2009) in a *P. edulis* forest at Nanjing, Jiangsu Province in China. At this site, many young *C. kiangsu* adults commonly feed on the leaves of small bamboo plants and various low-growing grasses. Tests were carried out only on sunny days during July 2008. Four hours after being immersed in aquous NaCl, four filter papers with or without decoys were placed in a line in random order on the low-growing grasses in the bamboo forest at a uniform distance of ten meters between each. Six hours later, the filter papers were collected, and the consumed areas were measured by placing the filter papers on squared paper and estimating the missing area in square millimeters. This procedure was replicated 20 times.

### Attraction/mortality tests

A preliminary experiment revealed that *C. kiangsu* adults can be captured by adding insecticide to aqueous NaCl bait in a container, and the locusts contacting the container would die inside of it or within 2 meters of it. Here we tested the effect of decoy conspecifics on attraction to aqueous NaCl containers, as determined by the number of dead individuals recorded (see below) in both the laboratory and in the bamboo forest.

Bisultap (an 18% aqueous solution) was diluted 600 times with a 3% NaCl aqueous solution. About 20 ml of the bisultap-contained solution was put into a white foam plastic container (15 cm in length, 10 cm in width and 2 cm in depth) and sloshed around so that the walls of the container were coated.

Four conspecific decoys, with male/female ratio of 1:1, were pinned on the outside surface of the walls of half of containers. The other half of the containers were left without decoys as controls.

In a non—choice test in the laboratory, containers differing in the presence or absence of conspecific decoys were placed individually in the small fabric cage. Four hours after dawn, one female was introduced in each test cage to avoid any female—female interactions. Each test was replicated 90 times. The number of dead adults was counted six hours later.

In the field experiment, attraction of the adults to the containers was tested from 28 July to 5 September, 2008, in a *P. edulis* forest at Zijing Mountains, Nanjing, Jiangsu Province in China. Four hours after the onset of photophase, four aqueous NaCl—treated containers differing in the presence or absence of conspecific decoys were placed in a line with random order on the low-growing grasses in the bamboo forest at a uniform distance of ten meters between each. Six hours later, the numbers of dead adults within a 2 m radius from the containers were counted. This procedure was replicated 20 times.

### Statistical analysis

Data are given as means ± SE. The differences between treatment and control were tested by Wilcoxon matched pairs test.

## Results

### Two-choice attraction test in the laboratory

**Figure 1.**
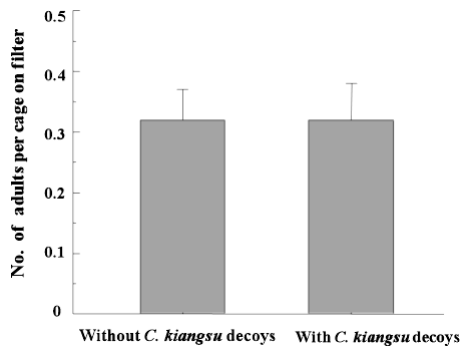
Mean numbers (± SEM) of *Ceracris kiangsu* females settling on white filter papers with and without *C*. *kiangsu* decoys (n=50) in a two choice test. The difference was tested by Wilcoxon matched pairs tests. High quality figures are available online.

Mean numbers of *C. kiangsu* females settling on plain filter papers (without NaCl) to which two conspecific decoys were attached, and control filter papers without decoys in small cages were calculated. The female adults showed no preference for control vs. treatment (*P*>0.1, Wilcoxon matched pairs test) (Figure 1).

### Feeding tests

**Figure 2.**
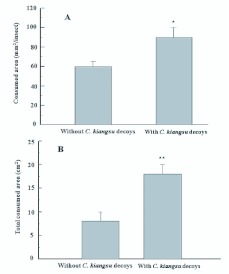
Feeding by *Ceracris kiangsu* adults measured by areas (± SEM) consumed of filter papers treated with NaCl aqueous solution (with or without decoys) in fabric screen cages (n=45) in the laboratory (A) or in the bamboo field (n=20) in Zijing Mountain, Nanjing, Jiangsu Province (B). * and ** indicated significantly different at *P*<0.05 and at *P*<0.01 compared by Wilcoxon matched pairs tests. High quality figures are available online.

In the laboratory, *C. kiangsu* female adults consumed more NaCl—treated filter papers to which 2 conspecific decoys were attached than those without decoys (*P*=0.001, Wilcoxon matched pairs test) (Figure 2 A). Similarly, in the bamboo forest, adults consumed significantly more NaCl aqueous solution-treated filter papers with 4 decoys present than those without decoys (*P*<0.001, Wilcoxon matched pairs test) (Figure 2 B).

### Attraction/mortality tests

**Figure 3.**
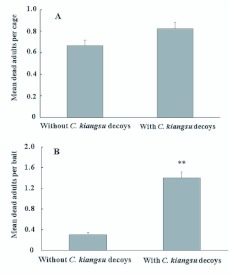
Mean dead *Ceracris kiangsu* adults (± SEM) killed by baits treated with bisultap differing in the presence or absence of conspecific decoys (2 females and 2 males) in fabric screen cages (n=90) in the laboratory (A) or in the bamboo field (n=20) in Zijing Mountain, Nanjing, Jiangsu Province (B). ** indicated significantly different at *P*<0.01 compared by Wilcoxon matched pairs tests. High quality figures are available online.

In the laboratory, containers treated with aqueous NaCl and bisultap with attached decoys attracted and killed 17.5% more females than containers without bisultap, but the difference was not significant (Figure 3 A, *P*>0.1, Wilcoxon matched pairs test). In the bamboo forest, however, the adults were more strongly attracted to and killed by containers treated with bisultap with attached decoys, compared to those without decoys (Figure 3 B, *P*<0.001, Wilcoxon matched pairs test).

## Discussion

Puddling substrates can be expected to be rare and difficult to find for insects (Downes 1973). One possible mechanism to improve an individual's chance finding puddling substrates is to search for conspecifics that may be puddling and join them. Joining behavior has been demonstrated in the context of puddling for several species of Lepidoptera, such as *Appias albino* (Gadagkar 2006), several Pieridae species (Beck et al. 1999), *Battus philenor* and *Papilio glaucus* (Arms et al. 1974, Otis et al. 2006), and have also been found in various ecological/behavioral contexts for some species of Hemiptera (Kidd 1976), Orthoptera (Muller 1998), Coleoptera (Raffa et al. 1993), Diptera (Barnhart and Chadwick 1953, Wiesmann 1962, Spivak et al. 1991, McCall and Cameron 1995, Collins and Bell 1996, Pinero and Prokopy 2004), and Hymenoptera (Fowler 1992, Reid et al. 1995, Raveret Richter and Tisch 1999, D'Adamo et al. 2000).

Olfactory and visual stimuli associated with puddling sites do not exist in isolation, because such sites are always subject to a complex of background signals. Accordingly, we tested the attractiveness of conspecifics to *C. kiangsu* adults in the laboratory and in a bamboo forest.

In the laboratory, *C. kiangsu* adults were not more attracted to untreated filter paper, with or without conspecific decoys. But they consumed significantly greater amount of NaCl—treated filter paper on which conspecific decoys were attached than those without decoys. In the bamboo forest, consumption of NaCl—treated filter papers was also significantly higher for those with decoys than those lacking decoys.

Moreover, in tests using containers treated with aqueous NaCl and the insecticide bisultap the presence of conspecific decoys attracted and killed significantly more adults in the bamboo forest than those without decoys. Since no other herbivorous insects were killed by NaCl bisultap—treated containers, we believe that *C*. *kiangsu* adults were the only species that consumed the NaCl—treated filter papers in the bamboo forest.

In contrast to the field experiments, the laboratory results showed that the presence of conspecific decoys did not significantly enhance the attractiveness to aqueous NaCl and bisultap—treated containers. The inconsistency may have resulted from the small space in laboratory tests. In a small fabric cage, *C. kiangsu* adults may easily find the treated containers irrespective of whether they had decoys or not. In nature, however, moving towards conspecific-occupied resources may facilitate foraging for NaCl and possibly other essential resources. An alternative explanation is that conspecific attractiveness could be confined to foraging contexts. If no puddling resources were available, no aggregation behavior occurred. Similarly, in *Vespula germanica*, the combination of meat and live conspecifics strongly enhances the attraction of conspecific foragers (D'Adamo et al. 2003; D'Adamo et al. 2004).

The cues that lead an individual to join conspecific group can be visual, chemical, and/or acoustical (Danchin and Wagner 1997; Prokopy et al. 2000; Despland 2001; Otis et al. 2006). In our experiments, we used dead decoys to rule out acoustical cues. During field experiments in bamboo forest, we observed that the adults were attracted to dead decoys over relatively large distance and from different directions. Therefore, we believe that visual cues assist *C. kiangsu* adults to find their conspecifics, although our experiments cannot completely rule out attraction of chemical cues from the dead decoys. Similar conclusions were reached by Beck *et al*. (1999) and Otis *et al*. (2006). Moreover, visual responses were partially responsible for congregation of conspecific gregarious locusts (Ellis 1959; Despland 2001).

In summary, our results demonstrated that moving towards conspecific-occupied resources may facilitate foraging of *C. kiangsu* adults for NaCl and possibly other essential resources. The presence of conspecifics may promote feeding on puddling resources. In the present paper, two decoys can attract *C. kiangsu* adults and enhanced feeding on NaCl solution immersed-filter paper in the laboratory. What is the effect of the number of decoys? Are there any sexual differences in joining behavior? More research is needed to answer these questions.
